# Efficient reduction method enables access to rare-earth telluride clusters

**DOI:** 10.1038/s42004-024-01178-3

**Published:** 2024-04-29

**Authors:** Chenyu Wang

**Affiliations:** Scientific Reports, https://www.nature.com/srep/

**Keywords:** Nanoparticles

## Abstract

Rare-earth telluride clusters enable the construction of highly crystalline rare-earth tellurides, but a general route for preparing such clusters is lacking. Now, a facile reduction approach produces rare-earth clusters supported by (poly)tellurido ligands, including a tri-tellurido ligand with a three-center, four-electron bonding structure.

Rare-earth tellurides (RE-Te) have transformed the quantum materials landscape with their unparalleled attributes, including charge density waves and magnetic semiconductivity. RE-Te clusters can serve as molecular precursors to bulk RE-Te materials with tunable compositions and high crystallinity. However, synthesizing these clusters is a significant challenge because lanthanide ions, which are hard Lewis acids, have a low tendency to bond with telluride-containing ligands, which are soft bases. Now, Zhiping Zheng, Jun Li and colleagues from Southern University of Science and Technology in China have developed a facile reduction approach that enables the quick production of tellurido ligands for coordination with a RE cluster core (10.1038/s44160-024-00511-x)^1^.

The clusters were prepared by mixing equimolar quantities of KCp* and anhydrous RECl_3_, followed by the successive addition of tellurium powder and KC_8_ (Fig. 1). “The use of a strong reducing agent is key,” explains first author Ding. “Our selected reductant, KC_8_, acts like scissors to fragment the Te powder into small units in solution (tellurido ligands), which are then captured by the RE cluster core formed in parallel. The introduction of Cp* (pentamethylcyclopentadienyl) as an electron-donating ligand effectively stabilizes the resulting RE-Te complexes, preventing them from decomposing,” he continues.

**Fig. 1:**
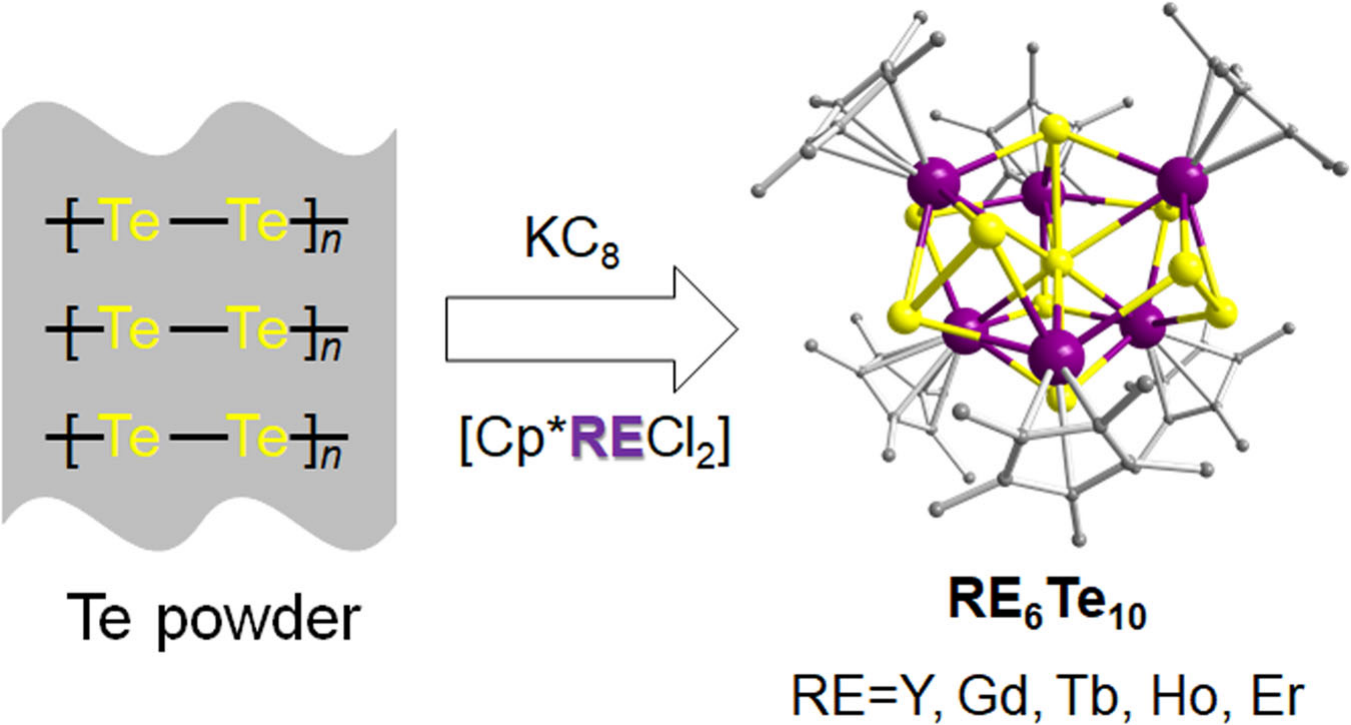
Illustration of the synthetic pathway to access RE-Te clusters. The Te powder is cleaved into (poly)tellurido motifs by a powerful reductant, KC_8_. As-generated Te ligands are quickly captured by simultaneously formed RE cluster cores. Cp* (pentamethylcyclopentadienyl), a typical electron-donating ligand, acts as a stabilizer for the resulting RE-Te complexes. Adapted from ref. ^1^.

“At the beginning of this project, we planned to prepare divalent RE complexes that could be used as reducing agents to reduce the tellurium powder. KC_8_ is one of the most powerful reducing agents, capable of reducing all RE^3+^ ions to RE^2+^. Isolating the divalent RE complexes is however very challenging, as they are extremely unstable. Therefore, we opted for a one-pot reaction instead, adding 2,2,2-cryptand to generate the single-crystalline compound [K(2,2,2-cryptand)]_2_[(Cp*RE)_6_(Te)_3_(Te_2_)_2_(Te_3_)],” shares Ding.

The team chose the Y_6_Te_10_ cluster as a representative subject for molecular and electronic structure characterizations. The cluster core was found to feature six Y atoms hinged upon a linear tri-tellurido motif, which in turn was characterized by quantum theoretical analyses as possessing four electrons delocalized over three atoms, forming a three-center, four-electron bond. Additionally, calculations indicate that the bond order for Te_3_^4-^ is significantly lower than that of a more common Te_2_^2-^ ligand, suggesting it is less stable in nature. The team hypothesized that fast reduction kinetics drive the production of the metastable Te_3_^4-^ ligand. “Rapid cleavage of bulk Te by KC_8_ can result in the formation of fragments with less stable electronic structures. The Te powder is initially reduced to tortuous Te_3_^2-^, and then quickly to the linear Te_3_^4-^. If the second reduction process is not swift enough, the Te_3_^2-^ will evolve into Te_2_^2-^ and Te^2-^,” comments Ding.

Moving forward, the group plans to further explore the potential of the rapid reduction principle for synthesizing RE-Te clusters supported by unexplored (poly)chalcogenido ligands. “We will also delve into the formation process of these RE-Te clusters in depth and use this understanding as a guideline to optimize our synthetic techniques. Additionally, we will employ these RE-Te complexes with tailored structures and compositions as precursors in the fabrication of highly crystalline RE-Te bulk materials with controllable phases,” envisions Ding.

## References

[1] Ding, Y. S. et al. Atomically precise semiconductor clusters of rare-earth tellurides. *Nat. Synth*. 10.1038/s44160-024-00511-x (2024).

